# Rationally designed *mariner* vectors for functional genomic analysis of *Actinobacillus pleuropneumoniae* and other *Pasteurellaceae* species by transposon-directed insertion-site sequencing (TraDIS)

**DOI:** 10.1186/s44149-021-00026-4

**Published:** 2021-11-26

**Authors:** Janine T. Bossé, Yanwen Li, Leon G. Leanse, Liqing Zhou, Roy R. Chaudhuri, Sarah E. Peters, Jinhong Wang, Gareth A. Maglennon, Matthew T. G. Holden, Duncan J. Maskell, Alexander W. Tucker, Brendan W. Wren, Andrew N. Rycroft, Paul R. Langford, on behalf of the BRaDP1T consortium, Duncan J. Maskell, Alexander W. ( Dan) Tucker, Sarah E. Peters, Lucy A. Weinert, Jinhong ( Tracy) Wang, Shi-Lu Luan, Roy R. Chaudhuri, Andrew N. Rycroft, Gareth A. Maglennon, Jessica Beddow, Brendan W. Wren, Jon Cuccui, Vanessa S. Terra, Janine T. Bossé, Yanwen Li, Paul R. Langford

**Affiliations:** 1 Section of Paediatric Infectious Disease, Department of Infectious Disease, Imperial College London, St. Mary’s Campus, London, UK; 2 Present Address: Wellman Center for Photomedicine, Harvard Medical School, Boston, USA; 3 Present Address: The Applied Diagnostic Research and Evaluation Unit, St George’s University of London, London, UK; 4 Department of Veterinary Medicine, University of Cambridge, Cambridge, UK; 5 Present Address: Department of Molecular Biology and Biotechnology, University of Sheffield, Sheffield, UK; 6 Department of Pathology and Pathogen Biology, The Royal Veterinary College, Hatfield, UK; 7 The Wellcome Trust Sanger Institute, Cambridge, UK; 8 Present Address: School of Medicine, University of St Andrews, St Andrews, UK; 9 Present Address: The University of Melbourne, Parkville, Victoria Australia; 10 Faculty of Infectious & Tropical Diseases, London School of Hygiene & Tropical Medicine, London, UK

**Keywords:** *Mariner*, Transposon, TraDIS, *Pasteurellaceae*, *Actinobacillus pleuropneumoniae*, *Pasteurella multocida*

## Abstract

Comprehensive identification of conditionally essential genes requires efficient tools for generating high-density transposon libraries that, ideally, can be analysed using next-generation sequencing methods such as Transposon Directed Insertion-site Sequencing (TraDIS). The *Himar1* (*mariner*) transposon is ideal for generating near-saturating mutant libraries, especially in AT-rich chromosomes, as the requirement for integration is a TA dinucleotide, and this transposon has been used for mutagenesis of a wide variety of bacteria. However, plasmids for *mariner* delivery do not necessarily work well in all bacteria. In particular, there are limited tools for functional genomic analysis of *Pasteurellaceae* species of major veterinary importance, such as swine and cattle pathogens, *Actinobacillus pleuropneumoniae* and *Pasteurella multocida*, respectively. Here, we developed plasmids, pTsodCPC9 and pTlacPC9 (differing only in the promoter driving expression of the transposase gene), that allow delivery of *mariner* into both these pathogens, but which should also be applicable to a wider range of bacteria. Using the pTlacPC9 vector, we have generated, for the first time, saturating *mariner* mutant libraries in both *A. pleuropneumoniae* and *P. multocida* that showed a near random distribution of insertions around the respective chromosomes as detected by TraDIS. A preliminary screen of 5000 mutants each identified 8 and 14 genes, respectively, that are required for growth under anaerobic conditions. Future high-throughput screening of the generated libraries will facilitate identification of mutants required for growth under different conditions, including *in vivo*, highlighting key virulence factors and pathways that can be exploited for development of novel therapeutics and vaccines.

## Background

*Actinobacillus pleuropneumoniae*, a member of the *Pasteurellaceae*, is the causative agent of porcine pleuropneumonia, a highly contagious, often fatal, respiratory disease that causes considerable economic losses to the swine industry worldwide (Sassu et al. 2018). Certain virulence factors have been shown to have specific roles in the pathogenesis of *A. pleuropneumoniae* infection including RTX toxins, capsule, lipopolysaccharide and various outer membrane proteins (Bossé et al. 2002; Chiers et al. 2010). In addition, two signature-tagged mutagenesis (STM) studies identified a large number of genes that contribute to the ability of *A. pleuropneumoniae* to survive and cause disease in pigs, though neither screen was saturating (Fuller et al. 2000a; Sheehan et al. 2003).

For more than two decades, Tn*10* has been the transposon of choice for generating libraries of random mutants of *A. pleuropneumoniae* for STM and other studies (Tascon et al. 1993; Rioux et al. 1999; Fuller et al. 2000a; Bossé et al. 2001; Sheehan et al. 2003; Grasteau et al. 2011). However, Tn*10* has an insertion site preference for GCTNAGC (Bender and Kleckner 1992), and different insertional hotspots were reported in *A. pleuropneumoniae* STM studies (Fuller et al. 2000a; Sheehan et al. 2003; Bossé et al. 2010), limiting the usefulness of this transposon for creating a fully saturating library. Clearly, a more random transposon mutagenesis system in *A. pleuropneumoniae* is required to allow genome-wide analysis of fitness using high-throughput sequencing methods such as Transposon Directed Insertion-site Sequencing (TraDIS) that not only precisely map, but also quantitatively measure the relative abundance of each transposon insertion in a pool of mutants (Gawronski et al. 2009; Langridge et al. 2009; van Opijnen et al. 2009).

The *Himar1* (*mariner*) transposon, originally isolated from the horn fly, *Haematobia irritans,* has been shown to insert randomly into the chromosomes of a wide range of bacteria with a dinucleotide target of “TA” (Picardeau 2010). In particular, *mariner* has been used for mutagenesis of *Haemophilus influenzae* (Akerley et al. 1998; Gawronski et al. 2009)*,* another member of the *Pasteurellaceae,* with a comparatively AT-rich genome, like *A. pleuropneumoniae*.

Preliminary (unpublished) investigations in our laboratory using the vector pMinihimarRB1 (Bouhenni et al. 2005), a kind gift from Dr. D. Saffarini, revealed that *mariner* is functional in *A. pleuropneumoniae*, however disadvantages including high background with kanamycin selection and retention of plasmid in initial transconjugants limited the usefulness of this vector for genome-wide analysis of fitness using TraDIS, a next generation sequencing method for mapping insertion sites (Langridge et al. 2009). We therefore decided to construct a *mariner* delivery vector incorporating the following desired components: a stringent selection gene carried by the mini-transposon; presence of DNA uptake sequences flanking the selection gene to allow for easy transfer of mutations between different strains *via* natural transformation; the presence of paired ISceI restriction sites just outside of the mini-transposon element to allow elimination of reads from residual plasmid during TraDIS; sequences to allow mobilization of the vector from a conjugal donor strain; and the C9 hyperactive mutant *mariner* transposase gene (Lampe et al. 1999), under control of either a constitutive or inducible promoter, encoded adjacent to the mini-transposon element to ensure stability of insertions. As the replication origin of the high copy number T-cloning vector, pGEMT, is the same as that in pBluescript, which we previously showed was not functional in *A. pleuropneumoniae* (Sheehan et al. 2000), it provided an ideal starting point for rational construction of a suicide *mariner* delivery system for use in *Pasteurellaceae* species.

## Results

### Construction and evaluation of *mariner* vectors

**Fig. 1:**
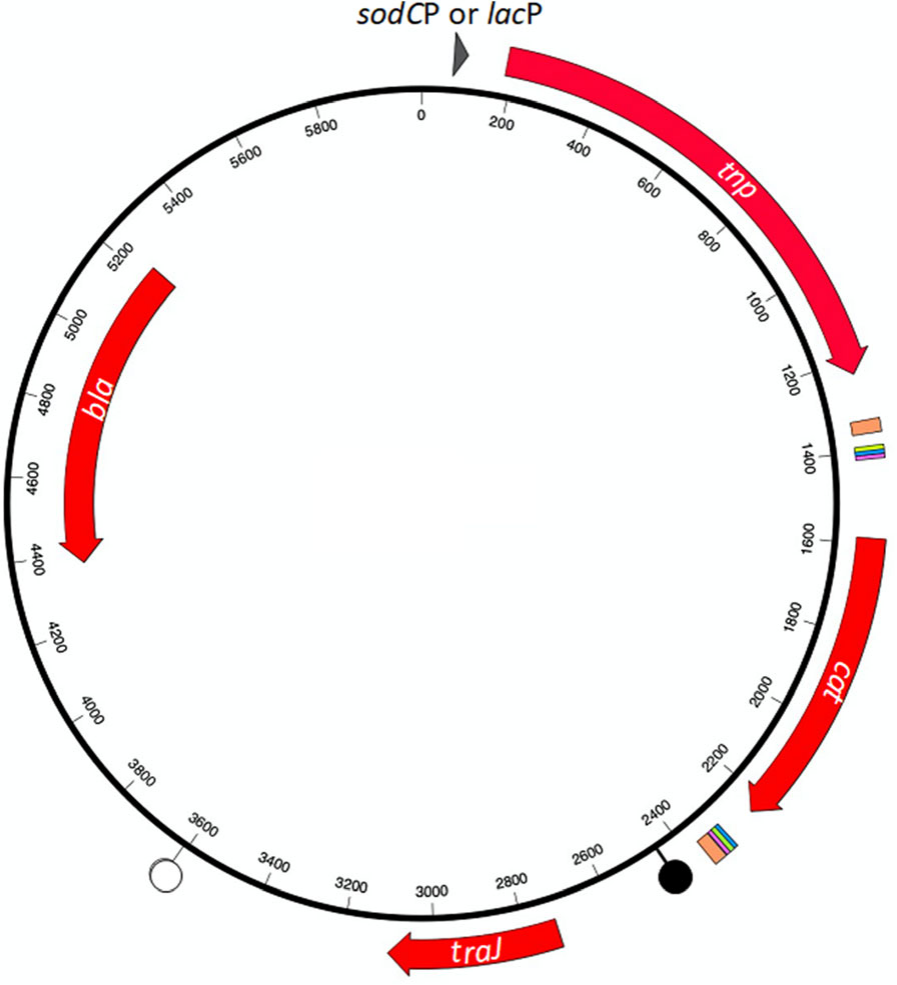
General map of *mariner* vectors, pTsodCPC9 and pTlacPC9, differing only in the promoter for the *tnp* gene. Genes *tnp* (*mariner* transposase C9 mutant), *cat* (chloramphenicol resistance), *traJ* (plasmid transfer gene), and *bla* (ampicillin resistance) are indicated by the appropriately labelled solid red arrows. The origin of plasmid transfer (*oriT*) is shown as a filled lollipop (), and the origin of plasmid replication (*colE1*) as a hollow lollipop (). Coloured blocks flanking the *cat* gene indicate the locations of *Himar1* inverted repeats (thick orange), and paired copies of DNA uptake sequences for *Neisseria* spp. (thin green), *H. influenzae* (thin pink) and *A. pleuropneumoniae* (thin blue). Arrowhead upstream of the C9 *tnp* gene indicates promoter sequences for either *A. pleuropneumoniae sodC* or *E. coli lac* genes, depending on the vector (pTsodCPC9 and pTlacPC9, respectively)

Two novel mobilizable *mariner* delivery vectors, pTsodCPC9 and pTlacPC9 (Fig. 1), differing only in the promoter driving expression of the C9 transposase gene, were successfully generated using pGEM-T as vector backbone. Conjugal transfer into *A. pleuropneumoniae* MIDG2331 from the diaminopimelic acid (DAP)-dependent MFD*pir*, achieved for both vectors, confirmed that *oriT* and *traJ* sequences were sufficient to facilitate mobilization. Comparison of results for *A. pleuropneumoniae* indicated that although the *sodC* promoter is constitutively expressed (Bossé et al. 2009), isopropyl-ß-D-galactopyranoside (IPTG) induction of C9 transposase expression from the *lac* promoter resulted in higher frequencies of transposition (10^− 6^–10^− 8^ compared to 10^− 7^–10^− 10^). Furthermore, similar frequencies of transposition were found in the genome of *Pasteurella multocida* MIDG3277 (10^− 6^–10^− 8^) using pTlacPC9, whereas no transposant was recovered using pTsodCPC9 in this species. Therefore, the pTlacPC9 vector was used for library construction in both *A. pleuropneumoniae* and *P. multocida*.

**Fig. 2:**
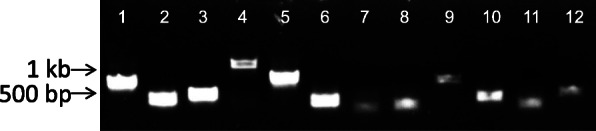
Linker PCR products for 12 randomly selected *A. pleuropneumoniae* mutants. Sequences flanking *mariner* insertions were amplified from AluI-digested linker-ligated DNA fragments using primers L-PCR-C and IR-Left_out (for amplification of the left flank). Lanes 1–12 show single amplicons for each of selected mutants, indicating the presence of a single insertion of transposon in each case

Colony PCR revealed that initial transconjugants retained extrachromosomal plasmid, as indicated by amplification of both chloramphenicol (Cm) cassette and *oriT*/*traJ* sequences (data not shown). Following subculture on selective agar, only the Cm cassette could be amplified from selected mutants, indicating loss of plasmid and integration of the transposon into the chromosome. Southern blot (not shown) and linker-PCR (Fig. 2) confirmed single insertions in different locations in 12 randomly selected mutants. Insertions were stable in the absence of selection for 20 generations.

### TraDIS analysis of *A. pleuropneumoniae* and *P. multocida mariner* libraries

**Fig. 3:**
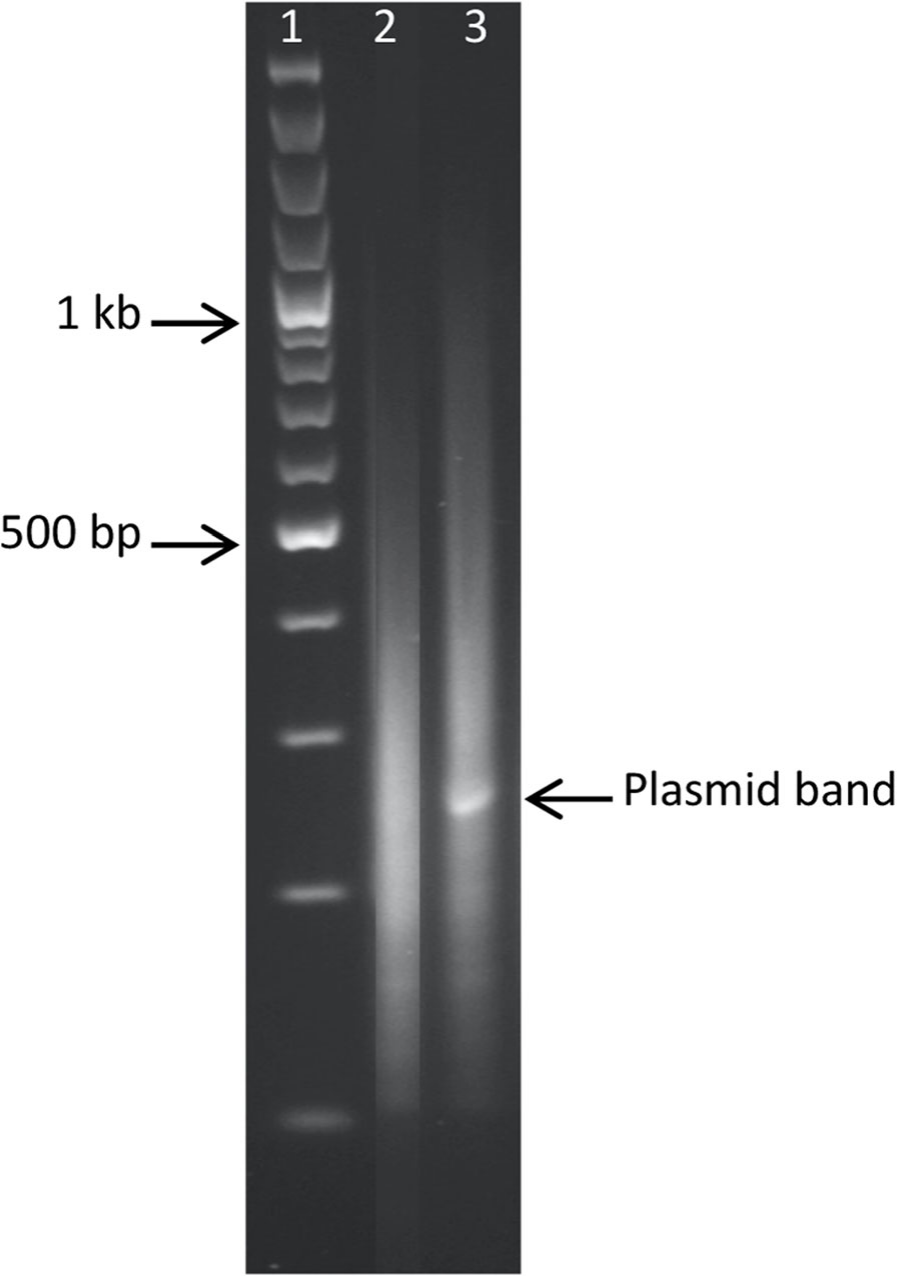
Comparison of linker PCR-products generated from pooled *A. pleuropneumoniae* library genomic DNA +/− ISceI digestion. Lanes: 1) 100 bp ladder; 2) ISceI-treated sample; 3) untreated sample. Amplification products were generated for the left flanking sequences, as in Fig. 2. The dominant plasmid band in the untreated sample is indicated

**Fig. 4:**
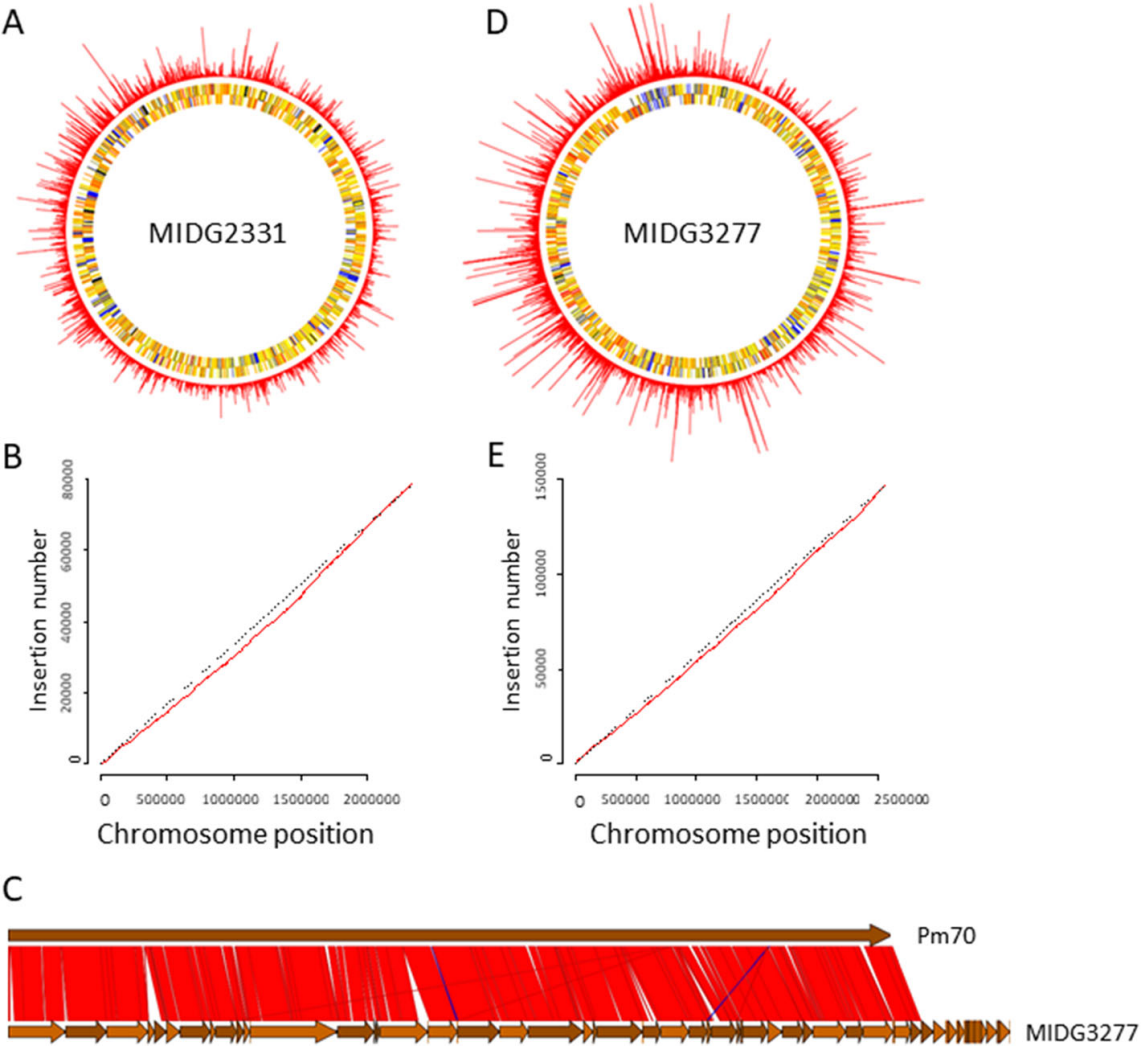
Distribution of *mariner* insertions identified in the *A. pleuropneumoniae* and *P. multocida* genomes. TraDIS reads for the respective pooled libraries were mapped to **A** the complete genome of *A. pleuropneumoniae* MIDG2331 (accession number LN908249); and **D** the draft genome (pseudochromosome) of *P. multocida* MIDG3277 (accession number ERZ681052). Each spike plotted around the chromosome represents a single insertion site, with the length of each spike proportional to the number of mapped sequence reads from that insertion site. A total of 78,638 unique insertion sites were identified in the *A. pleuropneumoniae* MIDG2331 library, and 147,613 in the *P. multocida* MIDG3277 library. In the MIDG3277 dataset, there were several insertion sites with large numbers of mapped reads. To enable insertions with fewer reads to be seen clearly, read coverage has been capped to a maximum of 50,000 in **D** (the true maximum coverage at an insertion site was 136,032 reads). The pseudochromosome of *P. multocida* MIDG3277 was assembled based on ordering of the draft sequence contigs following alignment, using NUCmer 4.0 (43), with the complete genome of *P. multocida* Pm70, as shown in **C**. Arrows indicate the position and orientation of the contigs. Red blocks indicate matches in the same orientation, blue blocks indicate matches in the reverse orientation. Plots of the cumulative insertion counts across the MIDG2331 chromosome **B** and MIDG3277 pseudochromosome **E** are shown in red, with a dotted line indicating the expected relationship for uniformly distributed insertions. Both libraries deviate from this, with a bias towards insertions close to the origin of replication, but insertions are found across the genome in both libraries

Analysis of linker-PCR products generated from DNA (+/− ISceI digestion) of the pooled *A. pleuropneumoniae mariner* library containing > 78,000 transconjugants showed a dominant plasmid-specific band only in the sample that was not treated with ISceI (Fig. 3). Both samples showed a strongly stained smear of DNA ranging from 100 to 500 bp in size, indicating a good distribution of insertions in the library. Subsequent TraDIS analysis of ISceI-digested *A. pleuropneumoniae* library DNA generated 16,565,883 sequence reads from the 5′ end of the *mariner* transposon. Of these, 15,381,053 (92.8%) were mapped to the complete MIDG2331 reference genome (Bossé et al. 2016), and 99.4% of those (i.e., 15,282,862 reads) corresponded to an insertion at a TA position. These mapped to 78,638 unique insertion sites, representing an insertion approximately every 30 bp on average and occupying 45.8% available TA sites in the genome. The insertion sites were distributed near randomly around the MIDG2331 genome (Fig. 4A), with the exception of a bias towards insertions closer to the origin of replication (Fig. 4B). This is likely due to those regions of the genome being present in multiple copies during transposition and is a commonly observed feature of transposon mutagenesis datasets (Langridge et al. 2009; Chao et al. 2016).

For analysis of the *P. multocida mariner* library, it was first necessary to generate a draft genome sequence for the isolate used MIDG3277. Sequencing on an Illumina HiSeq 2000 yielded 2,285,355 pairs of 100 bp sequence reads, which were assembled into 160 scaffolds, which were ordered based on a comparison with the *P. multocida* Pm70 genome (GenBank accession AE004439; Fig. 4C). The assembly had a total size of 2,563,460 bp, with an n50 of 77,141 bp. Contig annotation identified 2,490 coding sequences, together with 35 tRNA genes and 4 rRNA genes. A pseudochromosome sequence of MIDG3277 was obtained by concatenating the ordered scaffolds.

TraDIS analysis of ISceI-digested *P. multocida* library DNA generated 48,266,652 sequence reads from the 5′ end of the *mariner* transposon, of which 46,950,914 (97.3%) mapped to the draft genome sequence of MIDG3277. Of these, 99.7% (46,809,272 reads) corresponded to insertions at TA dinucleotides, at 147,613 unique insertion sites. These represent an insertion approximately every 17 bp on average, occupying 85.6% of available TA sites in the genome. The insertion sites were distributed near-randomly across the MIDG3277 pseudochromosome (Fig. 4D), with the exception of a replication cycle bias (Fig. 4E) similar to that observed for the *A. pleuropneumoniae* MIDG2331 library. The presence of this pattern despite the use of a draft reference genome provides independent confirmation that genomes of *P. multocida *Pm70 and *P. multocida* MIDG3277 are largely co-linear.﻿

### Preliminary screen for anaerobic mutants

Of approximately 5000 mutants each of *A. pleuropneumoniae* and *P. multocida* (from four different matings in both cases) screened for the presence of insertions in genes required for anaerobic growth, 19 and 15 mutants, respectively, were identified that failed to grow. Linker-PCR produced amplicons of different sizes that, when sequenced, mapped to unique TA dinucleotides in eight different genes in the *A. pleuropneumoniae* MIDG2331 genome (Table 1), and 15 different sites (some intergenic) in the *P. multocida* MIDG3277 genome (Table 2).

**Table 1:** Location of *mariner* insertions in anaerobic mutants of *A. pleuropneumoniae*

Gene	Product of disrupted gene	Gene location^a^	Location of insert^a^
*nhaB*	Na+/H+ antiporter protein	363852..365393C^b^	364160
*pflB*	Formate acetyltransferase	1178587..1180899C	1179054
			1179780
			1180383
*atpC*	ATP synthase epsilon chain	1922342..1922761C	1922665
*atpG*	ATP synthase gamma chain	1924188..1925054C	1924771
			1924970^c^
*atpA*	ATP synthase alpha chain	1925077..192661C	1925488
			1925791
			1925826^c^
			1926363
*atpB*	ATP synthase A chain	1928081..1928869C	1928204^c^
			1928575^c^
*fumC*	Fumarate hydratase	2051573..2052967C	2052243
MIDG2331_02098	D-alanyl-D-alanine carboxypeptidase	2163589..2164257C	2163863

^a^Location in the complete genome of MIDG2331 (accession number LN908249)

^b^Start and end positions of genes are indicated; those on the complementary strand are shown with the suffix C

^c^Insertions at these locations were mapped in two mutants each

**Table 2:** Location of *mariner* insertions in anaerobic mutants of *P. multocida*

Gene	Product of disrupted gene	Gene location^a^	Location of insert^a^
MIDG3277_1258	Predicted glycosyl transferase	contig21:104818..105990^b^	contig21:105340
*aroG*	Deoxy-7-phosphoheptulonate synthase	contig15:68162..69247	contig15:68149^c^
intergenic	Periplasmic pH-dependent serine endoprotease DegQ	contig 16:46775..48154	contig16:46700^d^
MIDG3277_1016	Hypothetical protein^e^	contig18:76828..77169C	contig18:77129
*aroA*	3-phosphoshikimate 1-carboxyvinyltransferase	contig18:79640..80962	contig18:80029
			contig18:80287
*sucC*	Succinate--CoA ligase subunit beta	contig3:86080..87246	contig3:86908
*ccmD*	Heme exporter protein CcmD	contig1:16084..16287	contig1:16136
MIDG3277_1814	F_0_F_1_ ATP synthase subunit I	contig31:36319..36726	contig31:36262^f^
*atpG*	F_0_F_1_ATP synthase subunit gamma	contig31:40520..41389	contig31:40497^g^
MIDG3277_0379	Hypothetical protein	contig6:168..512^h^	contig6:434
*pfeA*	Ferric enterobactin receptor	contig27:62820..64826C	contig27:64243
*menA*	1,4-dihydroxy-2-naphthoate octaprenyltransferase	contig23:97896..98801C	contig23:98875
*menB*	1,4-dihydroxy-2-naphthoyl-CoA synthase	contig23:34911..35768C	contig23:35383
*menC*	O-succinylbenzoate synthase	contig23:33645..34658C	contig23:34159

^a^Location in the draft genome of MIDG3277 (accession number ERZ681052)

^b^Start and end positions of genes are indicated; those on the complementary strand are shown with the suffix C

^c^Insertion located 13 bases upstream of the start of *aroG*; insertion likely disrupts transcription of *aroG*

^d^Insertion located 75 bases upstream of the start of *degQ* and 147 bases upstream of the start of MIDG3277_0890 encoding a putative NAD(P)H nitroreductase; insertion likely disrupts transcription of *degQ*

^e^This protein shares 100% identity with PM0836 (Genbank accession AAK02920) which was reported to be *in vivo* expressed (Hunt et al. 2001)

^f^Insertion located 57 bases upstream of the start of *atpI* (MIDG3277_1814); insertion likely disrupts transcription of *atpI* and remaining genes in the *atp* operon

^g^Insertion located 23 bases upstream of *atpG*; insertion likely disrupts transcription of *atpG* and remaining genes in the *atp* operon

^h^This predicted CDS is on a small contig and may be part of a larger gene (100% identity with the last 104 AAs of a 818 AA hypothetical protein P1062_0208970; Genbank accession ESQ71762)

## Discussion

The *mariner* transposon does not require host-specific factors for transposition and has a minimal requirement for TA dinucleotides as its target site, allowing for greater distribution in genomes compared to transposons such as Tn*5* and Tn*10*, which have “hot spots” for insertion. Tn*10* insertion at hotspots was a severe limitation in our previous *A. pleuropneumoniae* STM study (Sheehan et al. 2003; Bossé et al. 2010). Given the relatively AT rich genomes of *Pasteurellaceae* species, *mariner* should allow for creation of saturating mutant libraries in *A. pleuropneumoniae* and *P. multocida*.

Two novel mobilizable *mariner* vectors, pTsodCPC9 and pTlacPC9, were developed in this study. Both contain *mariner* mini-transposon elements that carry a Cm resistance gene, known to provide stringent selection of mutants in *A. pleuropneumoniae* (Bossé et al. 2014), flanked by paired DNA uptake sequences for each of *A. pleuropneumoniae*, *H. influenzae* and *Neisseria* spp. (Redfield et al. 2006; Treangen et al. 2008). Although initially designed for mutagenesis of *A. pleuropneumoniae*, all features included were designed to facilitate use in other Gram-negative bacteria. As it is often desirable to transfer mutations into wild-type strains to confirm the effect(s) of gene mutation, DNA uptake sequences were designed into the transposon to facilitate transfer of mutations by natural transformation in the species which selectively take up DNA containing these elements (Redfield et al. 2006; Treangen et al. 2008).

Restriction barriers can prevent or decrease efficiency of plasmid delivery by electroporation into various bacteria (Maglennon et al. 2013a; Luan et al. 2013), we therefore added *oriT* transfer origin and *traJ* genes from the broad host-range conjugative plasmid RP4 (Ziegelin et al. 1989) to facilitate vector delivery by conjugation. The presence of *oriT* alone on a plasmid is sufficient for mobilization by conjugal transfer machinery encoded by the *tra* operon (which includes *traJ*) present in the chromosome of a suitable donor strain, such as MFD*pir* used in the current study. However, as TraJ binds to the plasmid *oriT* to initiate plasmid transfer (Ziegelin et al. 1989) and also acts as a positive regulator for expression of the complete *tra* operon (Gubbins et al. 2002), a copy of this gene was included in the vectors, to enhance conjugation efficiency. Successful conjugal transfer of both vectors into MIDG2331 confirmed that these sequences were sufficient to allow mobilization from the DAP-dependent *Escherichia coli* strain MFD*pir* (Ferrieres et al. 2010). Use of MFD*pir* facilitates conjugation into wild-type recipient bacteria, instead of antibiotic resistant derivatives, as transconjugant selection is achieved on agar that does not contain DAP which is required for growth of the donor strain (Ferrieres et al. 2010).

The hyperactive C9 mutant transposase gene, previously shown to enhance *mariner* transposition efficiency (Lampe et al. 1999; Maglennon et al. 2013b) was placed under transcriptional control of either the *A. pleuropneumoniae sodC* promoter, which we have shown to be active under all conditions investigated so far, both in *A. pleuropneumoniae* and other *Pasteurellaceae* species (Bossé et al. 2009), or the *E. coli lac* promoter which allows induction by IPTG, and which is functional in a variety of bacteria including *A. pleuropneumoniae* (Sheehan et al. 2003). Although both resulting plasmids, pTsodCPC9 and pTlacPC9, were functional in *A. pleuropneumoniae*, the former generated a lower frequency of transposants. This may be due to over-production inhibition of expression from the highly active *sodC* promoter. This phenomenon was described in a recent study by Tellier and Chalmers (2020), where comparison of expression of the Hsmar1 *mariner* transposase from promoters of different strengths indicated the highest frequency of transposition was achieved with the weakest promoter. We also found that, although the *sodC* promoter was previously shown to function in *P. multocida* (Bossé et al. 2009), use of the pTsodCPC9 vector did not yield transposants in this species. In contrast, IPTG induction of C9 transposase gene expression from the *lac* promoter in the pTlacPC9 vector resulted in similar transposition frequencies in both *A. pleuropneumoniae* and *P. multocida*, indicating this vector will likely be a useful genetic tool for other bacteria.

Using the pTlacPC9 vector, saturating libraries were generated for both *A. pleuropneumoniae* (insertions on average every 30 bp) and *P. multocida* (insertions on average every 17 bp), with insertions randomly distributed around the respective chromosomes. In both cases, pooled mutant libraries were prepared from initial selective plate cultures of transconjugants (from multiple mating experiments) to limit selective expansion of clones. Under these conditions, extrachromosomal plasmid retained in the library can lead to high numbers of sequencing reads, interfering with TraDIS mapping. By engineering paired ISceI restriction sites (18 bp sequences not usually found in bacterial genomes) flanking the mini-transposon element in our vectors, we were able to effectively eliminate retained plasmid from the libraries prior to sequencing.

Both *A. pleuropneumoniae* and *P. multocida* are members of the *Pasteurellaceae* and are facultative anaerobes that infect the respiratory tracts of animals. Anaerobic growth is known to contribute to virulence of *A. pleuropneumoniae* (Jacobsen et al. 2004). However, little is known about the importance of genes contributing to anaerobic growth in *P. multocida*. In this study, we have screened a limited number of *mariner* mutants of both pathogens to identify genes important for anaerobic growth to further validate the randomness of insertions in these libraries and usefulness of our approach. Of the 19 *A. pleuropneumoniae* mutants sequenced, 15 unique insertions in eight different genes were identified (Table 1). Of 15 *P. multocida* mutants sequenced, all were in unique sites in the genome, with some insertions mapped to intergenic sites (Table 2). In both cases, mutants were selected from only four separate mating experiments, so it is possible that the four duplicate insertions mapped for *A. pleuropneumoniae* mutants were due to clonal expansion following insertion, even though mutants were randomly selected from the primary counter selection plate. As clonal expansion can skew libraries for over representation of more rapidly growing mutants, steps should be taken to limit this prior to TraDIS analysis. For example, multiple independent mating experiments, with shorter incubation periods during conjugal transfer, could be used to generate large libraries whilst minimising expansion of faster growing clones. Additionally, keeping the generated mutants in smaller, less complex pools could help further maximise representation of all fit mutants in a library (Chao et al. 2016).

For both organisms, the *atp* operon encoding the F_0_F_1_ ATP synthase appears to be essential for anaerobic growth. In *A. pleuropneumoniae*, 13 mutants had disruptions in four genes in *atp* operon, with nine unique insertion sites. In *P. multocida*, two of the anaerobic mutants had insertions that mapped to sites upstream of genes in the *atp* operon (56 bases upstream of *atpI*, the first gene in the operon, and 24 bases upstream of *atpG*). These intergenic insertions likely disrupt transcription of all, or part, of the *atp* operon, and therefore production of functional F_0_F_1_ ATP synthase. In the absence of oxidative phosphorylation, the F_0_F_1_ enzyme complex extrudes protons at the expense of ATP hydrolysis, to generate the driving force for solute transport and to maintain an acceptable intracellular pH value, and is required for function of certain enzymes involved in anaerobic respiration, such as formate hydrogenlyase (FHL) (Trchounian 2004). Furthermore, the activity of the FHL and F_0_F_1_-ATPase systems were found to facilitate the fermentative metabolism of glycerol in *E. coli* (Gonzalez et al. 2008). Maintenance of membrane potential by the hydrolytic activity of F_0_F_1_ ATP synthase has been shown to be vital for anaerobic growth of some bacteria when substrate level phosphorylation is the only source of ATP (Meyrat and von Ballmoos 2019). Although the *atp* operon has not previously been reported as essential for anaerobic growth of *A. pleuropneumoniae* or *P. multocida*, STM studies have identified genes of this operon as important for survival of these bacteria in the host during acute infection (Fuller et al. 2000a; Sheehan et al. 2003; Fuller et al. 2000b), where anaerobic growth is known to be essential.

An insertion in *nhaB*, encoding a Na^+^/H^+^ antiporter, was also found to be important for anaerobic growth of *A. pleuropneumoniae* in our current screen. As with the *atp* operon, *nhaB* has been shown to be required for *in vivo* growth of *A. pleuropneumoniae* (Baltes et al. 2007). The importance of interactions between proton and sodium cycles during both aerobic and anaerobic growth has been reported for certain pathogenic bacteria (Häse et al. 2001). Furthermore, Trchounian and Kobayashi (1999) demonstrated that *E. coli* grown anaerobically was more sensitive to increased extracellular Na^+^ concentrations than aerobically grown cells, especially at higher pH, and that mutants lacking a functional NhaA/NhaB encoded antiporter showed an even greater growth defect than wild-type strain under these conditions. The relative contributions of proton and sodium cycles to anaerobic growth of *A. pleuropneumoniae* and *P. multocida* warrant further investigation.

The importance of menaquinone biosynthesis for anaerobic growth of *P. multocida* was indicated by single insertions in each of *menA*, *menB,* and *menC*, as well as a single insertion three bases upstream of *aroG* (likely disrupting transcription), and two in *aroA*. Menaquinone is involved in anaerobic electron transport, and is derived from chorismate (pathway includes *menA*, *menB* and *menC*), which in turn is derived from shikamate (pathway includes *aroA* and *aroG*)*.*

Two of the *A. pleuropneumoniae* genes identified, *pflB* and *fumC*, encode proteins with known roles in anaerobic pathways. There were three separate insertions in *pflB* encoding pyruvate formate lyase which catalyses generation of formate* via* decarboxylation of puruvate. As mentioned above, the FHL complex is important in anaerobic respiration where it catalyses the conversion of formate into CO_2_ and H_2_ (Sawers 2005). A single insertion was mapped to *fumC* encoding fumarate hydratase, the enzyme responsible for catalysing conversion of malate to fumarate. Fumarate has previously been shown to be an essential terminal electron acceptor during anaerobic respiration in *A. pleuropneumoniae* (Jacobsen et al. 2004; Buettner et al. 2008).

Two identified *P. multocida* genes (*ccmD* and *sucC*) also have known anaerobic functions. Genes of the *ccm* operon are required for maturation of c-type cytochromes, including those involved in electron transfer to terminal reductases of the anaerobic respiratory chain with nitrate, nitrite or TMAO (trimethylamine-N-oxide) as electron acceptors (Schulz et al. 2000). The *sucC* gene encodes the beta subunit of succinate-CoA ligase, an enzyme in the aerobic citrate (TCA) cycle, where it catalyses hydrolysis of succinyl-CoA to succinate (coupled to the synthesis of either GTP or ATP). This enzyme also mediates the reverse reaction when required for anabolic metabolism, which can be particularly important under anaerobic conditions where the generation of succinyl-CoA *via* the oxidative pathway from 2-oxoglutarate is repressed (Mat-Jan et al. 1989; Shalel-Levanon et al. 2005).

The remaining mutants in both *A. pleuropneumoniae* and *P. multocida* have insertions in (or upstream of) genes not directly linked with anaerobic growth, however further investigation is warranted to determine their possible contributions.

## Conclusions

It is clear from this research that we have successfully constructed a *mariner* mini-transposon delivery vector capable of generating extremely large numbers of random mutants in *A. pleuropneumoniae*, *P. multocida*, and likely in other Gram-negative bacteria, which is amenable to genome-wide analysis of fitness using TraDIS. In preliminary experiments, we have established that the pTlacPC9 vector can be used in *Aggregatibacter actinomycetemcomitans* and *Mannheimia haemolytica*, though it remains to be determined if the generated libraries are saturating.

The limited number of anaerobic-essential genes (identified individually *via* phenotypic analysis in the current study) mapped to different insertion sites, including some which had not previously been associated with anaerobic growth of *A. pleuropneumoniae* and *P. multocida.* This suggests that TraDIS analysis of the pooled *mariner* libraries subjected to the same screen will identify many more genes with functions contributing to anaerobic fitness. In future work, we will screen our *mariner* libraries under different *in vitro* and *in vivo* growth conditions, broadening our understanding of conditionally essential genes in the *Pasteurellaceae*.

## Methods

### Bacterial strains and culture

For generation of *mariner* transposon libraries, we chose two different *Pasteurellaceae* species, *A. pleuropneumoniae* and *P. multocida*. The *A. pleuropneumoniae* clinical serovar 8 isolate, MIDG2331, was previously shown to be genetically tractable and has been fully sequenced (Bossé et al. 2016). The *P. multocida* isolate used in this study recovered from the respiratory tract of a calf in Scotland in 2008, and was shown to be sequence type 13, and part of clonal complex 13, in a multi-species multilocus sequence typing (MLST) study (Hotchkiss et al. 2011). This isolate, which has been labeled MIDG3277 in our collection, can be found in the pubMLST database (https://pubmlst.org/bigsdb?db=pubmlst_pmultocida_isolates) under the isolate name 22/4. *Pasteurellaceae* isolates were routinely propagated at 37 °C with 5% CO_2_ on Brain Heart Infusion (Difco) plates supplemented with 5% horse serum and 0.01% nicotinamide adenine dinucleotide (BHI-S-NAD). *E. coli* strains used were: Stellar [*F*^*−*^*, ara,Δ(lac-proAB) [Φ 80d lacZΔM15], rpsL(str), thi, Δ(mrr-hsdRMS-mcrBC), ΔmcrA, dam, dcm* and MFD*pir* [MG1655 RP4-2-Tc::[*ΔMu1::aac*(3)*IV-ΔaphA-Δnic35-ΔMu2::zeo] ΔdapA::(erm-pir) ΔrecA*] (Ferrieres et al. 2010). *E. coli* strains were maintained in Luria-Bertani (LB). Where appropriate, ampicillin (Amp; 100 μg/ml), chloramphenicol (Cm; 20 and 1 μg/ml for *E. coli* and *A. pleuropneumoniae*, respectively), and 0.3 mM DAP (required for growth of the MFD*pir* strain), were added to media.

### Genome sequencing, assembly and annotation

To obtain a draft genome sequence suitable for TraDIS analysis, genomic DNA was extracted from the *P. multocida* MIDG3277 strain using the FastDNA Spin kit (MP Biomedicals), and sequenced using an Illumina HiSeq 2000 at the Wellcome Sanger Institute. Illumina adapter sequences were trimmed from the reads using Cutadapt V. 1.8.1 (Martin 2011), and the trimmed reads were assembled using SPAdes V. 3.11.0 (Bankevich et al. 2012), using default parameters. The assembled contigs were aligned to the complete genome sequence of *P. multocida* Pm70 (GenBank accession AE004439) using nucmer V. 4.0.0 (Marçais et al. 2018). Using the alignment, contigs were reordered and reoriented to match the Pm70 reference genome, with one contig manually split where it overlapped the Pm70 origin. The reordered contigs were annotated using Prokka V. 1.11 (Seemann 2014).

### Construction of the *mariner* mini-transposon vectors

All primers are listed in Table 3. CloneAmp HiFi PCR Premix (Takara) was used to amplify sequences for cloning, and the QIAGEN Fast Cycling PCR Kit (Qiagen) was used for verification of clones, using the respective manufacturer’s protocols. When required, blunt PCR products were A-Tailed using 5 U Taq polymerase and 0.2 mM dATP prior to TA cloning into pGEM-T (Promega), according to the manufacturer’s protocol. Also, when required, DpnI digestion was used to remove plasmid DNA template from PCR products prior to cloning. All ligation products and In Fusion cloning products were transformed into *E. coli* Stellar cells (Clontech) by heat shock, according to the manufacturer’s protocol.

**Table 3:** Primers used in this study

Name	Sequence
CmDUSUSS_for	CGCGGATGCCGTCTG*AAGTGCGGT***ACAAGCGGT**CGGCAATAGTTACC
CmDUSUSS_rev	CGCG*AAGTGCGGT*ATGCCGTCTGA**ACAAGCGGT**TTCAACTAACGGG
CmDUSUSS_IRleft	CTGATAAGTCCCCGGTCTGCAGGCGGCCGCACTAGTGATTC
CmDUSUSS_IRright	CTGATAAGTCCCCGGTCTCGAAGTGCGGTATGCCGTCTG
Himar_IR	TAACAGGTTGGCTGATAAGTCCCCGGTCT
ISceI_left	***TAGGGATAACAGGGTAAT***CATGGCCGCGGGATTAACAGGTTG
ISceI_right	***ATTACCCTGTTATCCCTA***CGGCCGCACTAGTGATTAACAGG
M13_for	GTAAAACGACGGCCAGTG
M13_rev	GGAAACAGCTATGACCATG
oriTtraJ_left	_CCGCCTGCAGGTCGAC_AAAACAGCAGGGAAGCAGCGCTTTTC
oriTtraJ_right	_ACTCAAGCTATGCAT_GCATGGGGACGTGCTTGGCAATC
sodCPC9_left	_CGAATTGGGCCCGAC_CGCCAACCGATAAAACCTACATTTTGC
sodCPC9_right	_CCTCCTTTTCTAGTC_GCGGTACCGTCGACTGCAGAATTC
lacPC9_left	_CGAATTGGGCCCGAC_GTGAGCGCAACGCAATTAATGTGAGTTAG
lacPC9_right	_CCTCCTTTTCTAGTC_GGCGTAATCATGGTCATAGCTGTTTCC
C9sodC_left	_AGTCGACGGTACCGC_GACTAGAAAAGGAGGATTCCTCATATGG
C9lacP_left	_GACCATGATTACGCC_GACTAGAAAAGGAGGATTCCTCATATGG
C9_right	_CCGGGAGCATGCGAC_CCAGTGTGCTGGAATTCGCCCTTAGC
L-PCR-C	GATAAGCAGGGATCGGAACC
IR-Left_out	CACTTCAGACGGCATCCGCGAATC
IR-Right_out	GTTGAAACCGCTTGTTCAGACGGC

Specific DNA uptake sequences for *Neisseria* spp*.* (underlined), *H. influenzae* (italics), *and A. pleuropneumoniae* (bold) are indicated in the CmDUSUSS_for and CmDUSUSS_rev sequences

The ISceI restriction site is indicated in bold italic text

The 15 base overhangs for In Fusion cloning are shown in subscript text on the 5′ end of primers oriTtraJ_left to C9_right

A *mariner* mini-transposon encoding Cm resistance was constructed in stages using pGEM-T as vector backbone. Initially, a Cm cassette flanked by *A. pleuropneumoniae* uptake signal sequences (USS) was amplified from pUSScat (Bossé et al. 2014) using primers CmDUSUSS_for and CmDUSUSS_rev, which further added DNA uptake sequences for *Neisseria* spp*.* and *H. influenzae* on both sides of the cassette. The resulting 956 bp amplicon was A-tailed and cloned into pGEM-T yielding the plasmid pTCmDUSUSS. The *mariner* inverted repeat (IR) sequence (TAACAGGTTGGCTGATAAGTCCCCGGTCT) was then added to either side of the CmDUSUSS cassette in two subsequent rounds of PCR amplification and cloning into pGEM-T. In the first round, primers CmDUSUSS_IRleft and CmDUSUSS_IRright added the last 19 bases of the *mariner* inverted repeat to either side of the cassette. The full IR sequence was then used as a primer (Himar_IR) to amplify the transposon cassette prior to cloning into pGEM-T to yield pTHimarCm. Finally, paired I-SceI restriction sites were added to either side of the transposon by PCR using primers ISceI_left and ISceI_right, and the product was cloned into pGEM-T to yield pTISceHimarCm. The full insert was sequenced using M13_for and M13_rev primers prior to further modifications of the plasmid.

All further cloning steps were performed using the In Fusion HD cloning kit (Clontech), according to the manufacturer’s protocol. A sequence containing *oriT* and *traJ* gene was amplified from pBMK1 (Oswald et al. 1999), a generous gift from Gerald Gerlach, using primers oriTtraJ_left and oriTtraJ_right, and cloned into NsiI/SalI cut pTISceHimarCm to yield pTISceHimarCmoriT. The C9 hyperactive *Himar1* transposase gene (Lampe et al. 1999), amplified from pCAM45 (May et al. 2004) using primers C9_right and either C9sodC_left or C9lacP_left, was fused by overlap-extension (OE-PCR) PCR to either the *A. pleuropneumoniae sodC* promoter amplified from pMK-Express (Bossé et al. 2009) using primers sodCPC9_left and sodCPC9_right, or to the *lac* promoter amplified from pBluescript II KS (Agilent Technologies) using primers lacPC9_left and lacPC9_right. The sodCP-C9 and lacP-C9 OE-PCR products were cloned into ZraI cut pTISceHimarCmoriT to yield the *mariner* mini-transposon delivery vectors pTsodCPC9 and pTlacPC9, respectively. All inserts were confirmed by sequencing.

Purified pTsodCPC9 and pTlacPC9 plasmids were electroporated into *E. coli* conjugal donor strain MFD*pir* (Ferrieres et al. 2010), a generous gift from Jean-Marc Ghigo, with transformants recovered on LB containing 20 μg/ml Cm and 0.3 mM DAP.

### Bacterial mating and generation of *mariner* mutant libraries in *A. pleuropneumoniae* and *P. multocida*

Initially, the two different *mariner* mini-transposon delivery vectors, pTsodCPC9 and pTlacPC9, were evaluated for their ability to produce Cm-resistant mutants in *A. pleuropneumoniae* serovar 8 strain MIDG2331 following conjugal transfer from DAP-dependent *E. coli* MFD*pir* donor strain. For mating experiments, donor and recipient bacteria were grown separately in broth culture to an optical density at 600 nm (OD_600_) of approximately 1.0 (cultures were adjusted to equivalent OD_600_). Two hundred microliters of recipient strain were mixed with 0 to 200 μL donor strain (to give ratios of 1:1, 1:2, 1:4 and 1:8, as well as a control of recipient only). The bacteria were pelleted and re-suspended in 200 μL 10 mM MgSO_4_, and 20 μL aliquots were spotted onto 0.45 μm nitrocellulose filters (Millipore) placed onto BHI-S-NAD agar supplemented with DAP and, when required for induction of the *lac* promoter, 1 mM IPTG. Plates were incubated overnight at 37 °C with 5% CO_2_, after which bacteria were recovered in 1 mL sterile phosphate-buffered saline and 100 μL aliquots were plated onto BHI-S-NAD agar supplemented with 2 μg/mL Cm. In preliminary experiments, selected transconjugants were tested, both before and after subculture on selective agar, by colony PCR for the presence of the Cm cassette using primers CmDUSUSS_for and CmDUSUSS_rev, and for presence of plasmid backbone using primers oriTtraJ_left and oriTtraJ_right. Southern blot and linker-PCR using AluI-digested DNA were performed as previously described (Chaudhuri et al. 2009; Maglennon et al. 2013b) to confirm single random insertion of the transposon in selected transconjugants.

For *A. pleuropneumoniae mariner* library construction, a total of 14 conjugations were performed using the MFD*pir* + pTlacPC9 donor. For each mating, between 1,400 and 6,200 MIDG2331 transconjugants were collected from the selective agar plates and resuspended into respective 3 mL aliquots of BHI broth. Aliquots of each separate mating pool were stored as individual 2 mL freezer stocks containing a final concentration of 25% glycerol. A combined pooled library of transconjugants was generated by mixing equal 0.5 mL aliquots of each of the individual mutant pools prior to preparation of 2 mL freezer stocks containing a final concentration of 25% glycerol. All conjugations were performed at the same time and transconjugants were collected into pools directly from the selective agar plates, without further subculture, to avoid expansion of clones with increased fitness. Freezer stocks were stored at − 80 °C.

The pTlacPC9 vector was further assessed for the ability to generate Cm-resistant mutants in the related bacterium, *P. multocida.* In total, nine separate matings were performed using MFD*pir* + pTlacPC9 donor and *P. multocida* MIDG3277 recipient, with between 2,388 and 20,640 transconjugants selected per mating. Mutants were stored at − 80 °C as separate pools, and as a combined pool, in 2 mL aliquots in BHI broth containing a final concentration of 25% glycerol, as above.

### TraDIS analysis of the *A. pleuropneumoniae* and *P. multocida mariner* libraries

Genomic DNA was extracted from the pooled libraries of mutants using the FastDNA Spin kit (MP Biomedicals), according to the manufacturer’s protocol for bacterial cells. To assess the distribution of insertions in the pools prior to TraDIS, linker-PCR was performed as previously described (Maglennon et al. 2013b) with the exception that, for PCR amplification, primer L-PCR-C was paired with either IR_left_out or IR_right_out (see Table 3) in place of L-PCR-L or L-PCR-R, respectively. For comparison, 2.5 μg genomic DNA that were either untreated, or digested with ISceI to remove reads from residual plasmid, were used for linker PCR amplification of the left flank sequences, and the products were separated on 2% Nusieve agarose. Subsequently, 2 μg ISceI-digested DNA was used to prepare Illumina libraries for TraDIS analysis, as previously described (Luan et al. 2013).

TraDIS libraries were sequenced on an Illumina HiSeq 2500 at the Wellcome Sanger Institute. For the *A. pleuropneumoniae* library, TraDIS reads were mapped to the closed whole genome sequence of MIDG2331 (accession number LN908249). *P. multocida* TraDIS reads were mapped to the draft genome of MIDG3277 (accession number ERZ681052), constructed as described above. Reads were mapped using BWA mem (Li 2013) V. 0.7.17-r1188, with an increased penalty for 5′ clipping (−L 100,5). To reduce background noise, aligned reads which did not match a TA site at the 5′ end of alignment were excluded from further analysis using a custom Perl script, and the locations of insertions were extracted from the BAM file using SAMtools V. 1.13 (https://academic.oup.com/bioinformatics/article/25/16/2078/204688). Subsequent data analysis was performed using R V. 4.1.1 (https://www.R-project.org/).

### Preliminary screen for anaerobic mutants

Approximately 5,000 mutants each for *A. pleuropneumoniae* and *P. multocida* (from four separate mutant pools) were screened for insertions in genes required for survival during anaerobic growth. The mutant pools were plated on BHI-S-NAD supplemented with 2 μg/mL Cm at a density of 75 to 150 colonies per plate. Following overnight incubation at 37 °C with 5% CO_2_, colonies were transferred by replica plating onto two fresh selective plates. One plate was incubated with 5% CO_2_, and the other was placed in an anaerobic jar, at 37 °C overnight. Mutants that failed to grow anaerobically were re-tested to confirm the growth defect, and the site of transposon insertion was determined for each by direct sequencing of linker-PCR products.

## Data Availability

The complete sequences of the two *mariner* delivery vectors, pTsodCPC9 and pTlacPC9, have been deposited in GenBank under accession numbers MH644834 and MH644835, respectively. The plasmids are available upon request from the corresponding authors. The draft genome sequence of the porcine clinical respiratory isolate of *P. multocida*, MIDG3277, has been deposited in the European Nucleotide Archive (ENA) under the accession number ERZ681052, with the raw reads available under the accession number ERR200085. The raw TraDIS reads for *A. pleuropneumoniae* are available in ENA under the accession number ERR271132. The raw TraDIS reads for *P. multocida* are available in ENA under the accession numbers ERR744003, ERR744016, ERR752316, ERR752329, ERR755725 and ERR755738.

## Abbreviations

TraDIS: Transposon Directed Insertion-site Sequencing
STM: Signature-tagged mutagenesis
DAP: Diaminopimelic acid
IPTG: Isopropyl-ß-D-galactopyranoside
Cm: Chloramphenicol
FHL: Formate hydrogenlyase
TMAO: Trimethylamine-N-oxide
MLST: Multilocus sequence typing
BHI-S-NAD: Brain Heart Infusion supplemented with 5% horse serum and 0.01% nicotinamide adenine dinucleotide
LB: Luria-Bertani
Amp: Ampicillin
OD_600_: Optical density at 600 nm
ENA: European Nucleotide Archive
